# Albuminuria in patients with chronic obstructive pulmonary disease: a cross-sectional study in an African patient cohort

**DOI:** 10.1186/s12890-018-0694-5

**Published:** 2018-07-31

**Authors:** Festo K. Shayo, Janet Lutale

**Affiliations:** 1 Department of Internal Medicine, Muhimbili University of Health and Allied Sciences, P.O BOX 65001 Dar es Salaam, Tanzania; 2 Department of Internal Medicine, Muhimbili National Hospital, P.o box 14087 Dar es Salaam, Tanzania; 3 Tokyo Medical and Dental University, 1-5-45 Yushima, Bunkyo-ku, Tokyo 113-8510 Japan

**Keywords:** Cardiovascular disease, Albuminuria, Chronic obstructive pulmonary disease

## Abstract

**Background:**

Cardiovascular disease (CVD) is remarkably frequent in patients with chronic obstructive pulmonary disease (COPD). Albuminuria is a marker of vascular endothelial dysfunction and predictor of CVD events. Albuminuria is prevalent in patients with COPD as it has been shown in Caucasian and Oriental populations with COPD. The objective of this study was to determine the prevalence of Albuminuria and COPD severity correlates among black patients with chronic obstructive pulmonary disease in order to see whether a similar trend of albuminuria is also prevalent in this population.

**Methods:**

A total of 104 COPD patients were enrolled in the study. Lung functions were assessed by means of the Easy One™ spirometer. Albuminuria defined by urine albumin to creatinine ratio (ACR) was tested using CYBOW 12MAC microalbumin strips in a random spot urine collection. SPSS version 20 was used for data analysis.

**Results:**

In the studied population, 25/104 (24%) patients had albuminuria and 16/104 (15.4%) patients had CVD. Abnormal urine albumin (Albuminuria and Proteinuria) was present in all patients with CVD. In the subset of 46 COPD patients assessed for severity, 60.9% (95%CIs 46.1–73.9) had moderate COPD and 30.4% (95% CIs, 17.9–49.0) severe COPD. Albuminuria was moderately significantly associated with COPD severity, *p* = 0.049; (0.049 < *p* < 0.05). Participants who ever smoked cigarettes had significantly likelihood of severe and very severe COPD (OR 11.5; 95% CIs, 1.3, 98.4) however, the significance was lost when adjusted for age and gender.

**Conclusion:**

Albuminuria was prevalent in patients with COPD and it had a significant association with COPD severity.

## Background

The chronic obstructive pulmonary disease is currently the 4th cause of death worldwide; projected the 3rd by 2020 [1]. COPD has potential extra-pulmonary effects but it can be prevented and treated, however, no cure has been established. Cardiovascular disease is the most common extra-pulmonary presentation of COPD and therefore patients are at increased risk of morbidity and mortality due to acute cardiovascular events [2].

Different studies have shown that cardiovascular disease is common in COPD and likely to add to the complexity of the disease [3]. For instance; in the USA, the prevalence of cardiovascular disease in COPD patients was reported to be 22% versus 9% in non-COPD patients. In the UK, the relative risks of angina and myocardial infarction were 1.67 and 1.75, respectively, versus subjects without COPD [4]. A CONSISTE study in Spain showed that patients with COPD had a significantly higher prevalence of ischemic heart disease, cerebrovascular disease, and peripheral vascular disease compared non-COPD group [5].

Albuminuria is known to be a sensitive biomarker of endovascular dysfunction and a significant predictor of cardiovascular events and all-cause mortality in the general population. Vascular endothelial dysfunction is evident in patients with chronic obstructive pulmonary disease [6]. Presence of albuminuria indicates a state of generalized endothelial dysfunction and therefore it is a screening tool for early cardiovascular disease prevention [2]. Albuminuria is common in COPD patients and it independently correlates significantly with hypoxemia [2, 6, 7]. The aim of this study was to determine the prevalence of albuminuria and chronic obstructive pulmonary disease severity correlates in patients with COPD of African cohort. The results from this study will provide an insight on the prevalence of albuminuria in black population with COPD.

## Methods

### Study design and setting

**Fig. 1:**
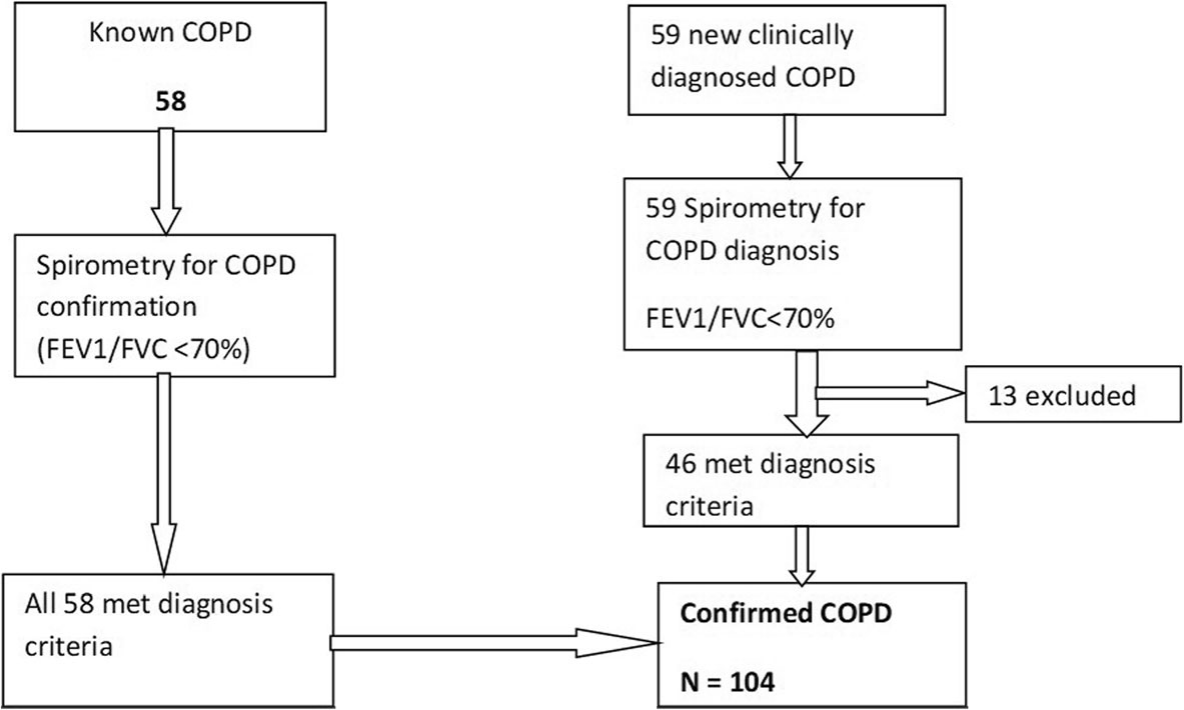
Flow Chart showing participants enrolment during the study

This was a hospital-based cross-sectional study. It was carried out in outpatient pulmonology clinic at Muhimbili National Hospital, Dar es Salaam, Tanzania from July 2016 to December 2016. Study participants were consecutively recruited from the Muhimbili national hospital pulmonology clinic. Patients with clinical diagnosis of COPD made by the attending physician/pulmonologist, underwent spirometry examination to confirm COPD diagnosis. A total of 117 patients were assessed for the study eligibility; of these, 58 were known COPD on follow up the clinic and 59 were new clinically diagnosed COPD patients. All known COPD patients underwent repeated spirometry without bronchodilator to re-confirm COPD diagnosis, of which all 58 were re-confirmed. The new clinically diagnosed COPD patients underwent both pre and post-bronchodilator to confirm COPD diagnosis, of which 46 were confirmed COPD diagnosis. Hence a total of 104 confirmed COPD cases were enrolled for the study (see Fig. 1). Inclusion criteria were (1) Patients with confirmed COPD defined FEV1/FVC < 70% of post-bronchodilator and pre-bronchodilator spirometry for new and previous cases respectively and (2) Age 18 years and above. Exclusion criteria were patients with urinary tract infection (UTI). The study was approved by the Muhimbili University of Health and Allied Sciences Senate Research and Publication Committee. The written consent was given by study participants.

A structured questionnaire was used to collect all important information from the study participants. These included a history of symptoms suggestive of COPD; progressive dyspnoea, chronic cough and chronic sputum production and history of cigarette smoking and biomass fuel exposure. Also, a history of any known associated co-morbidities like renal, cardiovascular disease, hypertension and malignancy condition were obtained. Baseline clinical parameters; blood pressure, respiratory rate, oxygen saturation and anthropometrics; body weight, height, and BMI were measured.

### Assessment of lung functions

Lung functions were assessed by means of the Easy One™ spirometer available at the clinic, manufactured by ndd Medizintechnik-Switzerland which complies with the 2005 American Thoracic Society/European Respiratory Society (ATS/ERS) spirometry standards and does not need daily calibration [8–10]. Spirometry was performed without a nose clip. Disposable mouthpieces (spirettes) were used and discarded after single use. COPD was classified as per 2015 GOLD guidelines.

### Assessment of albuminuria

The urinary albumin was screened using CYBOW 12MAC microalbumin strips in a random spot urine collection. These strips were available in a local market, manufactured by DFI CO., Ltd. 542–1 Daman Ri, Jinrye-Myun Gimhae-City Gyung-Nam Korea and have been approved by Food and Drug Authority (FDA). The reagent strip is a multi-strip for rapid determination of 12 components including protein, microalbumin, creatinine, nitrite, and leucocytes. CYBOW 12MAC reagent strips are both qualitative and semi-quantitative dip strips [11]. The earlier product of the strips (CYBOW 2MAC) has been used in some studies to assess for urinary albumin [12]. The sensitivity of this test ranged from 10 mg/L to150mg/L for microalbumin and 0.9 to 26.5 mmol/L for urine creatinine. Albumin-creatinine ratio (ACR) was then calculated to determine the level of albuminuria and expressed as mg/mmol. ACR < 2 mg/mmol for male and < 2.8 mg/mmol for female defined normoalbuminuria, ACR ≥ 2.5–29.9 mg/mmol for male and ≥ 3.5–29.9 mg/mmol for female defined albuminuria and ACR ≥ 30 mg/mmol for both male and female defined macroalbuminuria/proteinuria.

### Statistical analysis

The collected data were entered into EpiData 3.1 sheet and cleaned. Chi-square test and logistic regression were employed to ascertain the measure of statistical association by using SPSS version 20. The results were expressed as absolute numbers, mean plus or minus standard deviation (SD), and percentages. A *p*-value of < 0.05 was taken as statistically significant.

## Results

A total of 104 study participants were analysed. The mean age was 58.6 **±** 14.2 (SD) years, males constituted 56.7% of the study population. More than one-third of the study participants were of age range 42–83 years. More than half 56(53.8%) of study participants were smokers. Among smokers a large proportion 60.7% were heavy smokers; smoked > 10 pack year. A significant number of participants had a history of exposure to biomass fuel (firewood) and kerosene as cooking fuels; 40(38.5%) and 63 (60.6%) respectively, (Table 1).

**Table 1:** Social demographic characteristics of study participants (*N* = 104)

Variables		*n* (%)
Age groups (yrs.)		
28–41		11 (10)
42–55		33 (32)
56–69		35 (34)
70–83		22 (21)
> 84		3 (3)
Mean Age (SD)		58.63 ± 14.192
Sex		
Females		45 (43.3)
Males		59 (56.7)
Marital Status		
Single		10 (10)
Married		82 (79)
Divorced		1 (1)
Widow/Widower		11 (10)
Occupation		
Domestic/office work		40 (38.5)
Farmers/peasants		43 (41.3)
Industry		20 (19.2)
Others		1 (1.0)
Cigarette smoking:	Yes	56 (53.8)
Pack year	≤ 10 pack year	22 (39.3)
	> 10 pack year	34(60.7)
Biomass fuel exposure:	Firewood	40 (38.5)
	Charcoal	34 (32.7)
	Kerosene	63 (60.6)

A chronic cough and progressive dyspnoea were the most mentioned COPD related symptoms; 102/104 (98.1%) and 46/104 (44.2%) respectively. A small proportion 16/104 (15.4%) of the study participants had comorbid CVD by history. About one-third of the participants were overweight 32/104 (30.8%), and 12/104 (11.5%) obese. The proportion of study participants with elevated blood pressure on single reading was 39/104 (37.5%). (Table 2).

**Table 2:** Clinical characteristics of the study participants (*N* = 104)

Variables	Categories	*n* (%)
History suggestive of COPD	Progressive dyspnoea	46 (44.2)
	Chronic cough	102 (98.1)
	Chronic sputum production	13 (12.5)
History of Co-morbidities	Hypertension	1 (0.96)
	Cardiovascular disease	16 (15.4)
^b^Body Mass Index (BMI) in Kg/M^2^	Underweight (< 18.8)	10 (9.6)
	Normal weight (18.5–24.9)	50 (48.1)
	Overweight (25–29.9)	32 (30.8)
	Obesity (> 30)	12 (11.5)
^a^Blood pressure(mmHg): Systolic BP	Elevated (> 140)	21 (20.2)
Diastolic BP	Elevated (> 90)	18 (17.3)
Oxygen saturation %	Normal (92–100)	104 (100)

^a^BP is based on a single reading and therefore not diagnostic for hypertension

^b^Anthropometrics measured and categorized according to WHO

### COPD severity

Of the 46 COPD patients assessed for COPD severity; 60.9% (95%CIs 46.1–73.9) had moderate COPD and 30.4% (95%CIs, 17.9–49.0) severe COPD (Table 3).

**Table 3:** Post bronchodilator Spirometry assessing COPD severity (*N* = 46)

Variables (FEV1)	*n* (%)	95%CI
Mild (FEV1 ≥ 80%)	2 (4.3)	0–10.4
Moderate (50 ≤ FEV1 < 80%)	28 (60.9)	46.1–73.9
Severe (30 ≤ FEV1 < 50%)	14 (30.4)	17.9–49.0
Very severe (FEV1 < 30%)	2 (4.3)	0–14 7

### Predictors of COPD severity

In unadjusted regression model participants who ever smoked cigarette was significantly likely to have severe and very severe COPD (OR 11.5; 95% CI 1.3, 98.4; *p* < 0.05), however, the significance was lost when adjusted for age and gender. The participants who were; currently smokers, heavy smokers, and with a history of CVD had a non-significant increased the likelihood of severe and very severe COPD. (Table 4).

**Table 4:** Predictors of severe and very severe COPD among study participants assessed for COPD severity (N = 46)

Predictors	Test group	Comparative	OR	95% CI	*P* value
Smoking History	Ever smoker	Never smoker	11.5	1.3–98.4	0.026
Smoking status	Currently smoker	Former smoker	1.7	0.4–6.9	0.465
Pack-years smok.	≥10 - Heavy smoker	< 10- non-heavy	2.0	0.3–12.9	0.466
History of CVD	Yes	No	2.2	0.5–10.2	0.327

### Albuminuria

All 104 study participants underwent a dipstick urinalysis test using CYBOW 12MAC strips. Out of 104 participants 31 (29.8%) had proteinuria, 25 (24.0%) albuminuria, and 48(46.2%) normoalbuminuria. No urine sample had features of urinary tract infections. Therefore, the prevalence of albuminuria was 24%. Abnormal urine albumin (albuminuria and proteinuria) was prevalent in all study participants with CVD (Table 5).

**Table 5:** Prevalence of albuminuria (ACR) and its association with CVD among the study participants (*N* = 104)

		Urine albumin to creatinine ratio (ACR)			
		Normoalbuminuria	Albuminuria	Proteinuria	
		48 (46.2)	25 (24)	31 (29.8)	*p* < 0.001
		CI (36.2–55.5)	CI (17–33)	CI (21.5–37.5)	
History of CVD	Yes 16(100)	0 (0.0)	3 (18.8)	13 (81.2)	
	No 88(100)	48 (54.5)	22 (25.0)	18 (20.5)	

### COPD severity and albuminuria

Of the 46 participants assessed for COPD severity, albuminuria moderately significantly associated with COPD severity or with the lower level of FEV1% predicted, *p* = 0.049; (0.049 < *p* < 0.05). (Table 6).

**Table 6:** Association between albuminuria and COPD severity of the 46 participants assessed for COPD severity (*N* = 46)

VARIABLES	Total n (%)	Urine albumin to creatinine ratio (ACR)			
		Normal n (%)	Micro n (%)	Macro n (%)	*P*-value
Mild FEV1 ≥ 80%)	2 (4.3)	1 (50.0)	0 (0.0)	1 (50.0)	0.049
Moderate 50 ≤ FEV1 < 80%)	28 (60.9)	12 (42.9)	12 (42.9)	4 (14.3)	
Severe (30 ≤ FEV1 < 50%)	14 (30.4)	0 (0.0)	10 (71.4)	4 (28.6)	
Very severe (FEV1 < 30%)	2 (4.3)	0 (0.0)	2 (100.0)	0 (0.0)	
TOTAL n (%)	46	13	24	9	

## Discussion

This was a hospital-based cross-section study of 104 black Africans patients with COPD determining the prevalence of albuminuria and COPD severity correlates. Regarding COPD severity in the current study, a large proportion of study participants had moderate to severe COPD according to GOLD classification; 60.9% (95% [CI], 17.9–49.0] and 30.4, 95% [CI], 46.1–73.9) respectively. In a study by *Mehmood K* et al. the COPD was also classified according to GOLD criteria and the majority of study participants were in stage III (severe) and above; 55.7% [2]. This discrepancy can be accounted for by differences in study design and characteristics of study population between the two studies in respect to cigarette smoking. In the current study, the proportion of cigarette smoking was 53.8% while in the study by Mehmood K et al. all study participants were heavy smokers of >10pack year.

Albuminuria prevalence in the current study was 24% (95% [CI], 17.0–33.0) while abnormal urine albumin (albuminuria and proteinuria) was prevalent in all study participants with CVD. The presence of CVD was assessed through history alone. Of 104 study participants, 16/104 (15.5%) had CVD, of which 12 participants had the coronary arterial disease, 3 resolved stroke, and 1 hypertensive heart disease. More extra-pulmonary comorbidities could have been detected if laboratory markers were to be used, which was not the case in the current study**.** In a prospective cohort study conducted in India by *Mehmood K* et al. on albuminuria and hypoxemia in patients with COPD in 97 COPD smokers versus 94 non-COPD smokers as a controls over a period of 2 years; albuminuria was found to be more frequent in COPD smokers compared to smokers without COPD (20.6%versus 7.4% *p* = 0.007). The confounding co-morbidities like renal disease, diabetes, and cardiovascular disease were excluded using laboratory biomarkers and relevant history [2]. In the current study comorbid CVD, renal and diabetes were excluded by history only and hence might explain the differences in albuminuria prevalence.

The prevalence of albuminuria in the current study was similar to the prevalence of albuminuria found in a study done in Spain by *Ciro Casanova* et al. on albuminuria and hypoxemia in COPD patients (129 COPD cases versus 51 controls). In that study, the prevalence of albuminuria was 24% in patients with COPD and smoking history versus 6% in non-COPD smokers control; (*p* = 0.005), and all confounding co-morbidities were excluded using history and laboratory biomarkers [13].

In the current study, the COPD severity and albuminuria were significantly inversely related; the risk of albuminuria increased moderately significantly with COPD severity or the lower level of FEV1% predicted, (*p* = 0.049). All COPD patients with normoalbuminuria had GOLD stage I (mild) and II (moderate). A 12-year follow-up study in Norway by *Solfrid Romundstad* et al. on COPD and albuminuria in 53,129 patients showed that the risk for albuminuria increased significantly at lower levels of FEV1% predicted (*p* = 0.001). The majority (95.3%) of COPD patients without albuminuria had less severe COPD stages (GOLD stage of I and II) which is comparable to the findings in the current study [14].

Albuminuria in patients with COPD is also common in other associated co-morbidities including Chronic kidney disease (CKD), Pulmonary arterial hypertension (PAH), and atherosclerosis as a result of systemic endothelial dysfunction. Patients with COPD have shown to have endothelial injury pathways in the lungs and kidneys [15]. One study reported the evidence of the glomerular damage by increased ACR; (0.80 mg/mmol versus 0.46 mg/mmol) in COPD and non-COPD patients respectively [16].

Studies elsewhere have shown a significantly increased frequency of renal injury in COPD population compared to the non-COPD population. CKD comorbidity occurs frequently in COPD patients. For instance, one case-control study reported a significant CKD prevalence in COPD compared to non-COPD groups; (31% vs 8% *p* < 0.001) based on estimated glomerular filtration rate (eGFRCr), and (53% vs 15% *p* < 0.001) based on estimated cystatin C levels (eGFRCys). The odds ratio was 4.91 (95% CI, 1.94–12.46, *P* = 0.0008) and 6.30 (95% CI, 2.99–13.26, *P* = 0.0001) based on eGFRCr and eGFRCys respectively [17]. A systematic review and meta-analysis of observational studies reported increased odds of developing CKD (OR 2.20; 95% CI 1.83, 2.65) among COPD subjects compared to non-COPD subjects [18].

In this current study, the blood biomarkers for CKD, and other comorbidities were not carried out to ascertain their presence in COPD patients. A longitudinal study with thorough screening for these co-morbidities in order to explain their temporal association with COPD in the context of African patient cohort population is necessary, however.

This paper addressed the presence of albuminuria among African COPD patient cohort. COPD racial differences have not been well elucidated in the racial context. Literature is uncertain about COPD racial disparity due to differences in socioeconomic determinants risks for COPD. Limited available evidence reported that African-American with COPD is significantly younger and are less smoker. However, Africa-Americas have less emphysema than non-Hispanic Whites (NHW) but the same degree of airway disease. Furthermore, women of Africa-America ethnicity appear to be at higher risk of developing COPD than whites [19, 20].

It is common for people in low and middle-income countries including Africa to be exposed to biomass fuel/indoor pollution since childhood. Hence the mean age of COPD presentation in Africa is at the younger age some studies reported 35–45 years but also can be detected as early as the late teenage years. Women are the main cooks in the family and therefore are potentially vulnerable to particulate matters from biomass combustions. Young children less than 5 years of age do spend much time with their mothers hence are relatively equally exposed to biomass combustions [21–24]. Exposure to biomass fuel combustions is associated with increased prevalence of respiratory symptoms, reduced lung function and development COPD [25–27]. The domestic exposure to biomass fuel smoke in Tanzania and other African countries is alarmingly high. For instance, in Sub-Saharan Africa, the percentage of households using wood fuel varies from 86 to 99% in rural areas and 26–96% in urban areas. Overall 94% of African rural and 73% urban population used wood fuel (firewood and charcoal) as the primary source of energy [28].

Tanzania energy balance is dominated by biomass-based fuels, especially wood fuel (firewood and charcoal) which accounts for > 90% of primary energy supply [28]. More than 9 in 10 households (94%) use biomass fuels for cooking, heating, and lighting. Regarding tobacco use, less than 1 % of women (0.6%) smoke any tobacco while 14% of men smoke tobacco of which most of them smoke cigarettes on a daily basis [29]. Therefore, apart from tobacco smoking, the use of biomass fuels may be one of the important risk factors for COPD in Tanzania. The prevalence of COPD in Tanzania was conducted among 496 participants aged > 35 years in a rural setting by using spirometry diagnosis. Indoor and outdoor carbon monoxide (CO) levels from biomass fuel combustions were also measured. The overall prevalence of COPD was 17.5% of which 21.7% in males and 12.9% in females [30].

### Study limitations

The study had the following limitations; first, Chronic obstructive pulmonary disease severity classification was done only in newly diagnosed patients; this could underestimate it association with albuminuria in the context of sample size studied. For known COPD patients, it was difficult to stop their medications and arrange consecutively 2 days’ visits for assessing disease severity. Second, the use of history alone to describe the existence of comorbidity might have underestimated the existence of other conditions may cause albuminuria. A budget was not sufficient to cater for blood biomarker analysis.

## Conclusion

A large proportion of study participants who were assed for COPD severity had moderate and severe COPD. Albuminuria was prevalent in patients with chronic obstructive pulmonary disease and it increased significantly with COPD severity. All study participants with CVD had abnormal urine albumin (albuminuria and proteinuria). Screening for albuminuria in COPD patients can be used as an early marker of CVD risk and therefore prevention strategies can be planned. A longitudinal study to further explain the pattern of albuminuria among the black population is highly needed in order to have a better comparison with previous studies done among Caucasian and Oriental populations.

## Abbreviations

ACR: Albumin Creatinine ratio
ATS: American Thoracic Society
BMI: Body Mass Index
BP: Blood Pressure
CKD: Chronic Kidney Disease
COPD: Chronic Obstructive Pulmonary Disease
CVD: Cardiovascular Disease
eGFRCr: Estimated Glomerular Filtration Rate
eGFRCys: Estimated Cystatin C
ERS: European Respiratory Society
FEV1: Forced Expiratory Volume in 1 second
FVC: Forced Vital Capacity
MI: Myocardial Infarction
MNH: Muhimbili National Hospital
PAH: Pulmonary arterial hypertension
SPSS: Statistical Package for the Social Sciences
UK: United Kingdom
USA: United State of America
UTI: Urinary Tract Infections
